# Correlation of HER-2 over-expression with clinico-pathological parameters in Tunisian breast carcinoma

**DOI:** 10.1186/1477-7819-6-112

**Published:** 2008-10-22

**Authors:** Lobna Ayadi, Abdelmajid Khabir, Habib Amouri, Sondes Karray, Abdallah Dammak, Mohamed Guermazi, Tahya Boudawara

**Affiliations:** 1 Department of Pathology, Habib Bourguiba University Hospital, Sfax, Tunisia; 2 Department of Gynecology, Hedi Chaker University Hospital, Sfax, Tunisia; 3 Center of Biotechnology, Sfax, Tunisia

## Abstract

**Background:**

Breast carcinoma is a disease with a tremendous heterogeneity in its clinical behavior. Newer prognostic factors and predictors of response to therapy are needed. The aim of this study was to evaluate the expression of HER-2, estrogen receptor (ER) and progesterone receptors (PR) in breast carcinoma and to compare it with other prognostic parameters such as histological type and grade, tumor size, patients' age, and lymph node metastases.

**Patients and methods:**

This is a retrospective study conducted in the department of pathology at Sfax University Hospital. Confirmed 155 Cases of breast carcinoma were reviewed in the period between January 2000 and December 2004. We used immunohistochemistry to evaluate the expression of HER-2, ER, and PR receptor and Chi-square and Fisher exact test to correlate immunohistochemical findings with prognostic parameters for breast carcinoma such as patients' age, tumor size, histological type, histological grade and lymph node status.

**Results:**

The mean age of patients was 51.5 years, ranging from 22 to 89 years. 80 (51.6%) of the patients were below 50 years. The percentage of expression of HER-2, ER and PR was 26, 59.4, and 52.3%, respectively. HER-2 was over-expressed (3+) in 18.1% of the cases, was inversely related to ER expression (p = 0.00) and to PR expression (p = 0.048). This over-expression was also associated with a high tumor grade with marginal significance (p = 0.072). A negative correlation was noted between ER and PR expression and SBR grade (p = 0.000) and ER and age (p = 0.002).

**Conclusion:**

HER-2 over-expression was observed in 18.1% of Tunisian breast carcinoma affecting female patients. This group presents apparently an aggressive form of breast carcinoma with high histological grade and negative ER.

## Background

Breast carcinoma is the most common malignant tumor and the leading cause of carcinoma death in women with a tremendous heterogeneity in its clinical behavior. According to data from the Cancer Registry of Tunisia, breast carcinoma is the most frequent malignant neoplasm affecting Tunisian female patients with an incidence of 23.6/100.000 inhabitants [1]. The data of our registry show that women with breast carcinoma in Tunisia are relatively younger than in Western countries, with an average age of 51 years [2]. The incidence standardized on the age of the cancer of the breast in Tunisia was 16.7/100,000 women) [3]. This may suggest that breast carcinoma in Tunisia may have some biological features that need to be explored. Besides, the increasing incidence and significant breast cancer mortality (Overall survival rate = 50.5% after 5 years) [4] highlight the need for new therapeutic development, especially targeted treatment. A humanized monoclonal antibody, trastuzumab (Herceptin), targeting the human epidermal growth factor receptor 2 (HER-2) gene is a prime example of this new class of treatment. The (HER-2) gene is localized on chromosome 17q. It encodes for a transmembrane tyrosine kinase receptor protein that is a member of the HER family. HER-2 gene amplification is found in 10–34% of invasive breast carcinomas and is regarded as an important prognostic marker indicating poor patient survival [5]. The aim of this study was to evaluate the expression of HER-2, estrogen (ER) and progesterone (PR) receptors in breast carcinoma and to compare it with other prognostic parameters such as histological type and grade, tumor size, patients' age, and lymph node metastases.

## Patients and methods

### Patients and specimens

In this study, we conducted a comprehensive analysis of 178 breast carcinomas collected in the Sfax University Hospital between January 2000 and December 2004. Our study concerned a sample size of 155 because these were the only cases for which we had complete information about the patient and the tumor. Also, these were the only cases whose paraffin blocks had enough tissue to allow extra sections for our study and eventually for future examination. In situ carcinomas were not included in this study.

In fact, invasive breast carcinoma samples were studied after informed consent and IRB approval from the 155 patients. Patient characteristics are summarized in Table 1. The patients' age ranged from 22 to 89 years (mean age: 51.5 years), 80 (51.6%) patients were less than 50 years old and 72 (46.4%) were between the ages of 30 and 50.

**Table 1:** Clinicopathological features (n = 155)

	n	%
Age		
≤ 45 years	61	39,4
> 45 years	94	60,6
Tumor size		
≤ 50 mm	118	76,1
> 50 mm	37	23,9
Tumor grade		
1	17	11
2	98	63,2
3	40	25,8
Histologic type		
Ductal	130	83.8
Non ductal	25	16.1
Lymph node		
Negative	90	58,1
Positive	65	41,9
ER expression		
Negative	63	40,6
Positive	92	59,4
PR expression		
Negative	73	47,1
Positive	81	52,3
HER-2 status		
Negative (score 0, 1, 2+)	127	81,9
Positive (score 3+)	28	18,1
Surgery		
MRM	88*	81.4*
BCS	18*	16.6*
MWAC	2*	1.8*
Systemic therapy	92*	85.1*
Radiotherapy	76*	70.3*
Hormone therapy	46*	42.5*

HER-2: Human epidermal growth factor receptor 2; ER: Estrogen receptor; PR: Progesterone receptor; MRM: modified radical mastectomy; BCS: breast conserving surgery; MWAC: Mastectomy without axillary clearance. *: n = 108.

The tumor size varied from 0.9 cm to 16 cm (mean diameter: 4 cm). It was between 2 and 5 cm in 63.2% of the cases. The remaining cases had a tumor size more than 5 cm (23.8%) and less than 2 cm (12.9%). The histological type was determined on tissue sections. The microscopic grading of Scarff-Bloom-Richardson (SBR) was used: 17 cases were grade 1, 98 cases grade 2 and 40 cases grade 3. Lymph node involvement was detectable in 65% of cases. Patients were treated by a multimodality program: All patients underwent surgical treatment (modified radical mastectomy: 88 cases; breast conserving surgery: 18 cases; Mastectomy without axillary clearance: 2 cases). Systemic therapy, radiotherapy and hormone therapy (tamoxifen) were received in 92, 76 and 46 cases respectively.

### Pathological diagnosis

All surgical tissue specimens were fixed in 10% formaldehyde, embedded in paraffin, sectioned and stained with Hematoxylin/Eosin. According to the WHO classification [6], there were 130 (83.8%) ductal carcinomas and 25 (16.1%) non ductal carcinomas, subdivided into: 8 (5.8%) inflammatory carcinomas, 6 (3.8%) infiltrating lobular carcinomas, 5 (3.2%) mucinous carcinomas, 3 (1.9%) endocrine carcinomas, 1 (0.6%) medullary carcinoma, 1(0.6%) metaplastic carcinoma (carcinosarcoma) and 1(0.6%) oncocytic carcinoma.

### Immuno-histochemical staining

Immuno-staining of the HER-2 protein, estrogen (ER) and progesterone (PR) receptors was performed for all specimens. Four micrometer sections attached on silanized slides were de-waxed in xylene, rehydrated in graded ethanol and covered with 10 mM citrate buffer (pH 6). They were then incubated for 30 min with primary monoclonal antibodies against HER-2 (DAKO, clone 124, 1:100), ER (DAKO, clone 1D5, 1/25) and PR (DAKO, clone PgR636, 1/50), followed by incubation with biotin-labeled secondary antibodies. The streptavidin-peroxidase complex was visualized using di-aminobenzidine as a chromogenic substrate.

All slices were evaluated without knowledge of the clinical outcome. For each run of staining, a positive control slides were prepared from breast carcinoma known to be positive for the proteins studied. A semi quantitative score was used to record results of ER and PR staining according to the system established by Allred *et al *[7].

HER-2 was scored from 0 to 3 scales according to the criteria set by Dako. The staining was scored as negative (0) when no membrane staining was observed, or when membrane staining was observed in less than 10% of the tumor cells, weak positive (1+) if weak focal membrane staining was seen in more than 10% of the tumor cells, intermediate (2+) if weak to moderate, complete membrane staining was seen in more than 10% of the tumor cells; and strongly positive (3+) if intense and complete membrane staining with weak to moderate cytoplasmic reactivity was seen in more than 30% of the tumor cells. In the final analysis, only score 3 cases were considered as HER-2 overexpression cases. Fluorescence in situ hybridization (FISH) was not performed in this study.

### Statistical analysis

Statistical analysis was used to evaluate correlations between expression of HER-2, ER and PR and clinico-pathological parameters. It was done using the SPSS Inc. software (Version 13). Relationships between qualitative parameters were determined using the Chi-square and Fisher Exact Tests. Statistical significance was defined as p < 0.05.

## Results

### Relationships between HER-2, ER and PR expression

**Figure 1:**
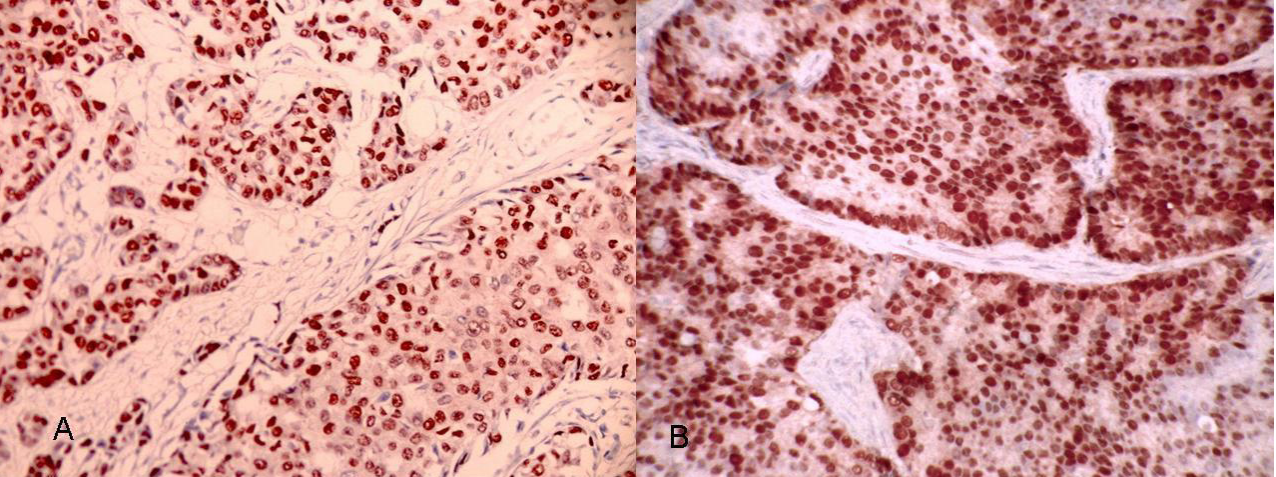
**Examples of strong nuclear immunostaining with hormonal receptors.** (a) ER+. (b) PR+.

**Figure 2:**
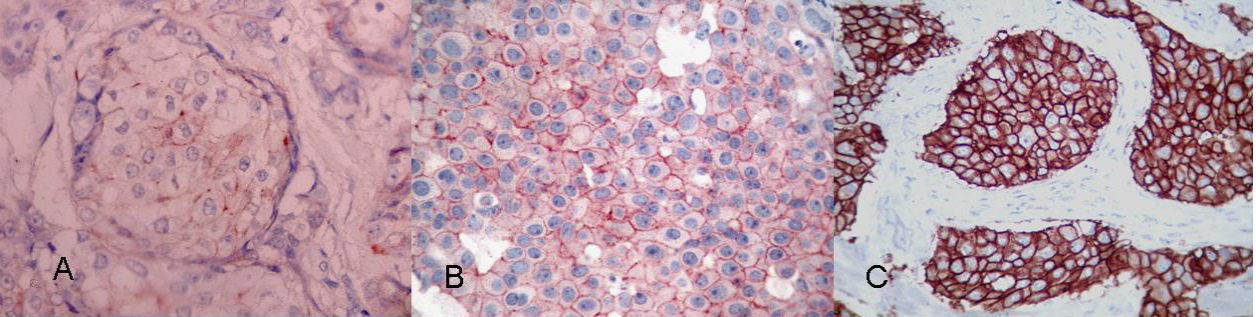
**Microscopy pictures illustrating the patterns of HER-2 immunostaining in breast carcinoma.** (a) Weak positive (1+) pattern exemplified by weak focal membrane staining seen in more than 10% of the tumor cells. (b) Intermediate (2+) pattern, showing weak to moderate complete membrane staining in more than 10% of the tumor cells. (c) Strongly positive (3+) pattern shows intense membrane staining with weak cytoplasmic reactivity in more than 30% of the tumor cells.

The expression rate of HER-2, ER and PR receptors was respectively 26, 59.4, and 52.3% (figure 1). HER2 was over expressed (3+) in 28 (18.1%) cases (figure 2). Simultaneous negative expression of ER, PR and HER-2 was found in 34 cases (21.9%). Conversely, Simultaneous expression of ER, PR and over expression of HER-2 was found in only 5 cases (3.2%). The percentage of HER-2 over-expression was sharply weaker in ER positive tumors: 7.6%, compared with 33.3% for ER negative tumors (p: 0.000). Over-expression of HER-2 was also inversely related to PR status (p: 0.048) (Table 2). Moreover, we have found a positive correlation between ER and PR (p = 0.000) (Table 3).

**Table 2:** Correlation between HER2 over-expression and Hormonal receptors status

	Her-2 over-expression	
	n (%)	p
ER		0.000
Positive	7 (7.6)	
Negative	21 (33.3)	
PR		0.048
Positive	10 (12.34)	
Negative	18 (24.65)	
ER/PR		0.000
Positive/Positive	5 (7.24)	
Negative/Negative	16 (32)	

HER-2: Human epidermal growth factor receptor 2; ER: Estrogen receptor; PR: Progesterone receptor.

**Table 3:** Correlation between ER and PR status (*P *= 0.000)

	RP		
	Negative	Positive	Total
RE			
Negative	50 (79,4%)	13 (20,6%)	63
Positive	23 (25,3%)	68 (74,7%)	91
Total	73	81	154

ER: Estrogen receptor; PR: Progesterone receptor

### Relationships between HER-2, ER and PR status and clinico-pathological parameters

In the comparative analysis of clinico-pathological parameters of breast carcinoma and HER-2 expression, the latter was not correlated with age nor with tumor size. Among patients with tumor size more than 5 cm (T3), 27% had HER-2 overexpression compared to 15.3% with tumors less than 5 cm (T1 and T2) (p: 0.104). In contrast, Patients with ER negative tumors were mostly young (< 30 years and between 30–50 years), as compared to positive ER expression in patients aged above 55 years. There was also a strong correlation with lymph node involvement (p = 0.000). HER-2 overexpression was also correlated with histological grade with marginal significance: only 14,8% of grade 1–2 carcinomas were HER-2 over expressed compared to 27.5% with grade 3 carcinoma (p = 0.072). A negative correlation between ER and PR expression and histological grade was noted (p = 0.000). There was no correlation between HER-2 status and histological subtype (ductal/non ductal). Inflammatory carcinoma overexpressed HER-2 in 50% of cases. All cases of mucinous carcinoma were HER-2 negative. No association was found between hormonal receptors expression and other pathological characteristics such as: histological type, tumor size, and lymph node involvement (Table 4). The only case of medullary carcinoma was triple negative (Table 5). Among tumors with ductal carcinoma SBR 1, only one case was HER-2+.

**Table 4:** Correlation of HER-2, ER and PR status with clinicopathological data

	HER-2 over-expression		ER Positive		PR Positive	
	n (%)	p	n (%)	p	n (%)	p
Age		0.28		0.002		0. 76
≤ 50 years	17 (21.3)		38 (47.5)		43 (53.8)	
> 50 years	11 (14.7)		54 (72)		38 (51.4)	
Tumor size		0.104		0.129		0.72
≤ 5 cm	18 (15.3)		74 (62.7)		63 (53.4)	
> 5 cm	10 (27)		18 (48.6)		18 (50)	
Histologic type		0.33		0.31		0.47
Ductal	22 (16.8)		80 (61.1)		70 (53.8)	
Non ductal	6 (25)		12 (50)		11 (45.8)	
Lymph-node		0.000		0.88		0.66
Negative	4 (4,4)		53 (58.9)		46 (51.1)	
Positive	24 (36.9)		39 (60)		35 (54.7)	
Tumor Grade		0.072		0.000		0.000
1–2	17 (14.8)		83 (72.2)		70 (61.4)	
3	11 (27.5)		9 (22.5)		11 (27.5)	

HER-2: Human epidermal growth factor receptor 2; ER: Estrogen receptor; PR: Progesterone receptor.

**Table 5:** Frequency of HER-2, ER and PR expression by histological subtype

**Histologic subtype**	**n (%)**	**ER+ (%)**	**PR+ (%)**	**HER2-+ (%)**
Ductal NOS	130 (83.3)	61.1	53.8	16.8
Inflammatory	8 (23,5)	37,5	37.5	50
Lobular	6 (17,6)	50	50	16.7
Mucinous	5 (14,7)	60	60	0
Endocrine	3 (8,8)	66.7	33.3	33.3
Metaplastic	1 (2,9)	100	100	0
Medullary	1 (2,9)	0	0	0
Oncocytic	1 (2,9)	0	0	100

NOS: not otherwise specified;HER-2: Human epidermal growth factor receptor 2; ER: Estrogen receptor; PR: Progesterone receptor.

We have studied the lymph node involvement comparing ER+PR+HER-2+ with other ERPRHER-2 subgroups; but we don't found any statically significant correlation (Table 6).

**Table 6:** Frequency of HER-2 expression for lymph-node status by joint ER/PR expression

	No			N+		
	HER-2			HER-2		
	Negative	Positive	Total	Negative	Positive	Total
ER- PR-	27(90%)	3 (10%)	30	7 (35%)	13 (65%)	20
ER- PR+	7 (100%)	0 (0%)	7	1 (16.7%)	5 (83.3%)	6
ER+ PR-	14 (100%)	0(0%)	14	7 (77.8%)	2 (22.2%)	9
ER+ PR+	38 (97,4%)	1(2.6%)	39	25 (86,2%)	4 (13,8%)	29
Total	86 (95.55)	4(3.45)	90	40 (62.5)	24 (37.5)	64

N0: without lymph node involvement; N+: with lymph node involvement; HER-2: Human epidermal growth factor receptor 2; ER: Estrogen receptor; PR: Progesterone receptor.

## Discussion

The prognosis of breast carcinomas is related to a large variety of clinical and pathological factors. It is well known that ER, PR and HER-2 represent the most acceptable factors for predicting prognosis response or resistance to treatment and the potential use of newer drugs [8-11]. The association between Her-2 gene amplification and poor prognosis was first determined in 1987 by Slamon *et al *[12]. In the present study, HER-2 over expression was seen in 18.1% of Tunisian female patients' breast carcinomas. It has been reported that 10–34% of breast carcinomas over-express the HER-2 receptor. This characteristic is associated with more aggressive tumor behavior. The ErbB receptors themselves regulate estrogen-signaling pathways, either by directly phosphorylating the estrogen receptor, or by activating mitogen-activated protein kinases, which in turn enhance estrogen receptor signaling [13,14]; furthermore, previous studies of breast cancer cell lines have implicated growth factor in signaling repression of PR expression [15].

80 (51.6%) patients were less than 50 years old and 72 (46.4%) of them were between the ages of 30 and 50. In contrast to what is commonly known about a rising incidence of breast cancer with age, our results showed that 51.6% of the patients examined were young with an age below 50 years. The mean age of these patients was 51.5, and 46.4% of them were between the ages of 30 and 50. This age distribution is significantly younger than what is currently seen in Western and Arab countries [16,17], and requires further careful examination to determine the nature of the predisposing factor(s). One possible explanation is that traditional marriages among first-degree relatives in Tunisia are very common, and, accordingly, hereditary factors could play a major role. Another factor could be the degree of obesity associated with a diet high in fat, carbohydrate, and protein, and lack of exercise, which have been prevalent in Tunisia the two last decades. The relative young mean age of our patients may be explained by the age distribution in our population or by risk factors that may be particular to our country.

Some authors [18-20] have suggested that HER-2 overexpression is associated with young age; our study failed to reveal a significant relationship between HER-2 overxpression and patient's age (women older or younger than 50 years). This may be due to that our report is a small series.

In contrast, we found that there is a significant association between the nature of the tumors' expression of ER and the age of the patients. These results are in agreement with most reports in the literature which show an association between the expression of ER and age in breast carcinoma [18,19,21,22]. However, other studies have found no association between the age and the degree of expression of ER by the tumor [23].

In our study, we did not find any significant association between the age of the patients and their tumor expression of PR. Similar findings were reported by many authors [24-26]. Other studies, however, have reported a higher tumor expression of PR in patients older than 59 years, as compared to those between 50 and 59 years [27].

Our results showed a tendency of HER-2 overexpression to be more associated with larger tumor size although this difference was not statistically significant. Similarly, the fraction of tumors larger than 5 cm tended to have higher rates of HER-2 overexpression than those below 5 cm in size (27% versus 15.3%). Several studies have found no association between Her-2 overexpression and tumor size [18,28-30]. On the other hand, there was no statistically significant correlation between hormonal status and size. This result is in concordance with data reported in the literature [31,32].

In our study, the most common histological subtypes were ductal-not otherwise specified (83.8%), followed by inflammatory carcinoma (5.1%), lobular carcinoma (3.8%) and mucinous carcinoma (3.8%). Inflammatory breast carcinoma is particularly common in Tunisia and the region of North Africa [24]; this confirms the relatively high percentage of occurrence of this variety in our study. The highest percentage of HER-2 overexpression (50%) was seen in inflammatory carcinoma; this support the view that it known to have an aggressive clinical course, very often resulting in early recurrence and death. However, no statistically significant correlation was found between HER-2 overexpression and histological type which confirm the data of many reports [33,34]. In contrast, recent reports suggest that HER-2 overexpression is significantly more likely in infiltrating ductal carcinomas than in infiltrating lobular carcinomas [35]. Our results show that there was no significant difference between ductal and lobular carcinoma regarding HER-2 overexpression. This could imply that, although lobular carcinoma is less common than ductal carcinoma, lobular carcinoma could be equally aggressive. On the other hand, there was no statistically significant correlation between hormonal status and histological type which has also been described in the literature [31,32]. In our study, some limitations should be considered when interpreting the results. This study was limited by relatively small numbers of non ductal subtypes: endocrine, medullary, metaplastic, and oncocytic carcinomas were three or fewer, resulting in estimates with wide confidence limits.

Most studies have correlated HER-2 overexpression with poor histological or nuclear grade of the primary tumor [9-11,28-30,36-38], whereas others have not [39,40].

Traina *et al*. [41] have demonstrated that only HER-2 (3+) and histopathologic grading 3 are significantly associated with overall survival. Similarly, our study showed that tumors with grade 3 were more often HER-2 negative.

In this study, we also found that lower grade (1–2) of the tumor was significantly related to the expression of ER and PR. Similar findings were reported by many studies [42,31,32].

The prognostic importance of HER-2 has also been analyzed in the context of patient subgroups with or without lymph node involvement. Most of the studies that have examined the prognostic role of HER-2 in patients with positive lymph nodes have shown that HER-2 amplification/overexpression is associated with a worse outcome in either univariate or multivariate analysis [37,41,43,44]. This finding was also confirmed in our study. A few studies, however, have not shown statistically significant correlation between HER-2 and lymph node status [18,28,30].

Despite the great variation in levels of HER-2 positivity, nearly all investigators report a negative relationship between HER-2 status and steroid receptors levels [8-11,15,18,28-30,36-38,41]. Our results confirm this data. This inverse association has been linked to the fact that estrogens and its receptor are required to suppress HER-2 [13,14]. This leads to lower or absent hormone receptors in women with HER-2 positive breast cancers. This is one of the reasons why women who express HER-2 may be resistant to tamoxifen [45].

## Conclusion

Analysis of HER-2 status in breast carcinoma is important because it provides valuable prognostic, predictive and therapeutic information. In this study about 155 patients with infiltrating breast carcinomas, HER-2 overexpression was evaluated by immunohistochemistry and was observed in 28 cases (18.1%). Our results showed that HER-2 over-expression correlates with aggressiveness parameters such as high histological SBR grade, lymph node metastases and negative ER/PR status.

## Abbreviations

HER-2: Human epidermal growth factor receptor 2; ER: Estrogen receptor; PR: Progesterone receptor.

## Competing interests

The authors declare that they have no competing interests.

## Authors' contributions

LA designed the study, interpreted the results of HER-2, ER and PR expression, and drafted the manuscript. AK reviewed the histopathology of breast carcinoma, graded all the cases histologically and helped to draft the manuscript. HA participated in the sequence alignement and helped to draft the manuscript. SK prepared the histological slides and helped the immunoperoxydase stains on the cases. AD reviewed all clinical data and helped to draft the manuscript. MG participated in the design of the study and performed the statistical analysis. TSB conceived the study and participated in the design of the article and coordination and helped interpretation of the results of HER-2, ER and PR expression. All authors read and approved the final manuscript.
